# Supplementary material for: Non-catalytic motor domains enable processive movement and functional diversification of the kinesin-14 Kar3
Source: eLife. 2015 Jan 27;4:e04489. doi: 10.7554/eLife.04489 (PMC4338441; doi:10.7554/eLife.04489)
Supplement: Supplementary file 1. — This file contains a detailed description of the mathematical model for cooperative Cik1–Kar3 kinesin movement. DOI: http://dx.doi.org/10.7554/eLife.04489.023 [file elife04489s001.docx]

**1. Supplementary File 1**

**Mathematical Model of the Cooperative Movement of Cik1-Kar3 heterodimers.**

**Table S1. Model Parameters**

| $V$ | Average velocity of movement. |
| --- | --- |
| $\sigma_{v}$ | Standard deviation of the velocity. |
| $F_{stall}$ | Stall force of a Cik1-Kar3 heterodimer. |
| $k_{stiff}$ | Stiffness of the linkage between heterodimers. |
| $k_{ON}$ | Binding rate of Cik1-Kar3 heterodimers to microtubules. |
| $k_{OFF}^{o}$ | Unbinding rate of Cik1-Kar3 heterodimers from microtubules at zero loads. |
| $k_{OFF}^{MAX}$ | Maximal increase of unbinding rate due to applied force. |

**Table S2. Model Variables**

| $x_{i}$ | Coordinate of the i-th Cik1-Kar3 heterodimer along the microtubule axis. |
| --- | --- |
| $v_{i}$ | Velocity of the movement of i-th Cik1-Kar3 heterodimer. |
| $v_{i}^{o}$ | Velocity of the movement of i-th Cik1-Kar3 heterodimer at zero load. |
| $f_{i}$ | Net force applied to i-th attached heterodimer. |
| $k_{OFF}^{i}$ | Unbinding rate of i-th Cik1-Kar3 heterodimer |
| $n$ | Number of Cik1-Kar3 heterodimers in a team |

**Description of the Model**

Unless stated otherwise the MT is positioned horizontally with its minus end pointing to the right and all movement occurs only along one axis. We consider a single heterodimer as a solid unit, which binds to a microtubule at the rate $k_{ON}$, moves at the rate $v_{i}$ and unbinds at the rate $k_{OFF}^{i}$. When the Cik1-Kar3 dimer binds to a microtubule its moving rate is drawn from the normal distribution of rates and then considered to be constant until the next unbinding/binding event: $v_{i}^{o}\sim N(V,\sigma_{v})$. In order to model the case of multiple dimer interactions with the microtubule we assumed that in a team of Cik1-Kar3 heterodimers each dimer is connected to the other via a spring that is characterized by a stiffness $k_{stiff}$. Further, we assumed that the movement of dimers occurs independently and according to the velocities assigned to each dimer upon binding (Figure 1 - figure supplement 3).

The tension generated by the above velocity differences in the linkage between the dimers applies an external force to them. It is currently not known for the Cik1-Kar3 kinesin how applied force affects the rate of movement and binding-unbinding rate. The overall tendency, based on studies of conventional and some other kinesins, can be assumed to be that a hindering force would slow down the kinesin and increase the unbinding rate. In order to take this effect into account we assume a simple dependency of the velocity and the dissociation constant on the applied force based on available data (Schnitzer, Visscher, and Block 2000; Thorn, Ubersax, and Vale 2000; Okada, Higuchi, and Hirokawa 2003; Block et al. 2003). For the velocity we assume a linear force-velocity relationship given by

$v_{i}=v_{i}^{o}\left(1+\frac{f_{i}}{F_{stall}}\right)$ (1)

with negative $f_{i}$ implying a hindering force applied to the heterodimer.

For the dissociation rates we assume


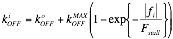
 (2)

These dependencies are illustrated in Figure 1 - figure supplement 3B,C.

**Mathematical Simulation of Movement**

We use the following algorithm for modeling the movement of kinesin teams. First in a team consisting of $n$ independent heterodimers a random dimer is attached to the microtubule. Next a loop is initiated, which updates the positions and the states of all dimers. The loop executes four consecutive actions:

1. The positions of all attached heterodimers are updated based on their velocities and applied forces. Increment of the position of i-th dimer is given by: $dx_{i}=v_{i}\cdot dt$
2. Based on the new positions the forces are updated. The force acting on i-th attached heterodimer is given by: $f_{i}=\sum_{j} k_{stiff}(x_{j}-x_{i})$, where summation goes over all attached dimers.
3. For each unattached dimer we calculate the following expression:

$t_{k}^{ON}=-\frac{\ln(1-r_{k})}{k_{ON}}$;

where$r_{k}$ is a random number from uniform distribution on the interval (0,1): $r_{k}\sim U(0,1)$

The state of the dimer is switched from detached to attached at current the iteration when $t_{k}^{ON}<dt$. When implemented over many iterations this algorithm yields exponentially distributed attachment times with on-rate $k_{ON}$. The bound position of the new dimer is assigned as:

$x_{k}=r_{k}(\max\left\{x_{i}\right\}-\min\left\{x_{i}\right\})+\min\{x_{i}\}$, where again $r_{k}\sim U(0,1)$ and $\left\{x_{i}\right\}$ are positions of all attached dimers.

1. Same algorithm is implemented for the detachment. Detachment time is calculated using:

$t_{k}^{OFF}=-\frac{\ln(1-r_{k})}{k_{OFF}^{k}}$; where $k_{OFF}^{k}$ is given by (2) and $r_{k}\sim U(0,1)$

State of a dimer is switched to detached when $t_{k}^{OFF}<dt$ .

The average position of the team at each step is calculated as $X=<x_{i}>$ for all attached dimers. The loop stops when there are no more attached dimers. Using this simulation the run length and the velocity are calculated for each trial. The simulation is repeated $10^{3}$ times. All trials are pooled to determine the average run length, the average velocity and their corresponding standard deviations. The simulation is repeated for different number of kinesins in a team and different model parameters. An example of the simulation for a team consisting of 3 kinesins is shown in Figure 1 - figure supplement 3D.

The most straightforward way to fit theoretical curves to the data is to use least-squares fit. This becomes more difficult when separate datasets such as velocity, run length and their standard deviations need to be fitted simultaneously. Instead we introduce an objective function that can be calculated across different datasets (velocity, run length and standard deviations) and minimized simultaneously to find the best fit. We use relative difference between experimental and theoretical data across all comparable variables defined by the following relation:

$m=\frac{2}{N}\sum_{i} \sum_{j=1}^{4} \frac{\left|p_{i,j}-q_{i,j}\right|}{\left|p_{i,j}+q_{i,j}\right|}$ (3)

, where $p_{i,j}$ and $q_{i,j}$ are theoretical and experimental values of the parameters. Indexes $j$ and $i$ enumerate size of the complex and type of the parameter, respectively. $N$ is the total amount terms under the sums. Table 3 shows notations for the indexes:

**Table S3. Notation of the theoretical parameters used in (3). Experimental parameters $q_{i,j}$ run through the same indexes.**

|  |  | j=1 | j=2 | j=3 | j=4 |
| --- | --- | --- | --- | --- | --- |
| i=1 | average velocity | $p_{1,1}$ | $p_{1,2}$ | $p_{1,3}$ | $p_{1,4}$ |
| i=2 | standard deviation of the velocity | $p_{2,1}$ | $p_{2,2}$ | $p_{2,3}$ | $p_{2,4}$ |
| i=3 | run length | $p_{3,1}$ | $p_{3,2}$ | $p_{3,3}$ | $p_{3,4}$ |
| i=4 | standard deviation of the run length | $p_{4,1}$ | $p_{4,2}$ | $p_{4,3}$ | $p_{4,4}$ |

Theoretically we can simulate teams of any size, but since there is no reliable statistics for complexes larger than four kinesins, under our experimental conditions, we limited all our simulations by complexes of size four, therefore j runs only until 4. When i and j both change from 1 to 4, total number of terms in (3) is 16: $N=16$ . By minimizing objective function (3) we find optimal parameters that approximate experimental data best. The lower the value of the objective function at the minimum the better is the fit. Its absolute value can be interpreted as an average deviation of the theory from experiment across all parameters. That is *m*=0.1 means that theory doesn’t deviate from the experiment more than 10% on average across different datasets.

**Parameter Estimation**

Some parameters in our model are completely defined based on experimental data that we have. The rest of the parameters are unknown. We varied unknown parameters in order to find the optimal fits. Ranges in which certain parameters can be varied we estimated based on the available literature. Table 4 summarizes values and ranges used for different parameters in our model.

**Table S4. Values and ranges of parameters used in simulations.**

| Para-meter | Value or Range used in simulations | Description |
| --- | --- | --- |
| $V$ | 50 nm/sec | Based on the experimental measurement of single Cik1-Kar3. |
| $\sigma_{v}$ | 20 nm/sec | Based on the experimental measurement of single Cik1-Kar3. |
| $F_{stall}$ | 0.1 – 6 pN | Stall force of conventional kinesins has been found to be ~ 6pN ([4](#_ENREF_4), [5](#_ENREF_5)). We expect this to be an upper estimate because absence of one of the catalytic domains most likely reduces the maximal force that can be generated by kinesin. Stall force was also measured for single headed diffusive kinesins and was found to be ~ 0.15 pN ([3](#_ENREF_3)). This is likely to be at the lowest possible value. |
| $k_{stiff}$ | 0.03 pN/nm | Tail of Cik1-Kar3 kinesin consists mostly of coiled-coil somewhat similar to that of conventional kinesins. Compliance of the conventional kinesin is known from optical trapping experiments and numbers vary between 0.01 – 0.5 pN/ nm ([5](#_ENREF_5), [6](#_ENREF_6)). While keeping all other parameters constant we verified that variation of the $k_{stiff}$ within this range does not affect quality of the fit (Figure 1 - figure supplement 3E,F). Therefore we typically use constant value of $k_{stiff}=0.03$pN/nm in our simulations. |
| $k_{ON}$ | 0 - Inf | No specific range was used for this parameter. |
| $k_{OFF}^{o}$ | 0.088 sec^-1^ | This parameter was calculated from experimental data based on the inverse average lifetime of the single Cik1-Kar3 on microtubules. |
| $k_{OFF}^{MAX}$ | 0 - Inf | No specific range was used for this parameter. |

**Theoretical Results**

There are published models that study cooperative movement of kinesins. While some of them have been published recently (Berger et al. 2012; Zhang 2011), the model that fully studies movement of many kinesins per team has been published in 2005 (Klumpp and Lipowsky 2005). Before modeling more complex behavior we asked whether this model can reasonably fit our data. Our model described above is reduced to (Klumpp and Lipowsky 2005) by assuming $\sigma_{v}=0$; $k_{OFF}^{MAX}=0$; $F_{stall}\to\infty$, which we call a simplified model. This model has only one free parameter, $k_{ON}$, which can be easily fit to experimental data. It yields
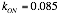
sec^-1^ and value of the objective function $m=0.19$. This model fits the experimentally observed average velocities and a run length well, but does not describe the observed decrease in standard deviation of the velocity as a function of the number of kinesins (Figure 1 - figure supplement 3G). The main disadvantage of this model, however, is that by introducing variable rates for kinesins it allows distance between extreme dimers in a team to increase without any restriction. Since there is no dependence of the velocity and unbinding rate on the applied force there is efficiently no mechanism for synchronization of the movement between kinesins in a team. Our simulations show that for a complex of size 4, the distance between extreme dimers in a team varies up to 900 nm due to variability of the individual rates. The average size of fluctuation across 1000 simulations is 150 nm. One would expect that such large variations in the overall size of the moving team should at least occasionally occur in experimental trials which however we never see experimentally.

Next we used the more general model to fit our experimental data. In this model elastic compliance between heterodimers in a team exerts forces on moving dimers. Since the velocity and the off-rate are dependent on the force, this effectively allows for synchronization of the movements between individual dimers. A fast moving dimer will move forward and stretch the linkage to those lagging behind. This in turn will exert a hindering force on the faster moving dimer and leads to its slow-down. For a slow moving dimer the opposite situation will lead to its speed-up due to the assisting force from the rest of the attached dimers.

This model with an elastic linkage between the dimers has three independent unknown parameters $F_{stall}$, $k_{ON}$ and $k_{OFF}^{MAX}$. While there are many ways how the data can be fitted using these three parameters the simplest approach is to fix one or two parameters at constant values, and allow the other to vary parametrically. We have chosen three different values of $F_{stall}$= 0.2, 1 and 4 pN and for each of them varied $k_{ON}$ and $k_{OFF}^{MAX}$ simultaneously to find the best fit. For each value of $F_{stall}$ we were able to find a pair of ($k_{ON}$,$k_{OFF}^{MAX}$) that fits the data well (see Table 5 and Figure 1 - figure supplement 3H,I) as judged by the value of the objective function at the minimum shown in the rightmost column in the Table 5.

**Table S5. Two-parameter fits of the experimental data for different values of $F_{stall}$.**

| $F_{stall}$ (pN) | $k_{ON}$ (sec^-1^) | $k_{OFF}^{MAX}$ (sec^-1^) | Value of the objective function at the minimum |
| --- | --- | --- | --- |
| 0.2 | 0.57 ± 0.1 | 3.7 ± 1.2 | 0.1 |
| 1 | 0.55 ± 0.1 | 4.0 ± 1.3 | 0.08 |
| 4 | 0.49 ± 0.09 | 5.3 ± 2.3 | 0.1 |

The variability of the distance between extreme dimers in a team consisting of 4 dimers does not exceed 50 nm for the smallest possible spring stiffness studied ($k_{stiff}=0.01$ pN/nm). This means that the same images should be observed on the camera irrespective of the actual number of dimers present in a team as their distance between the dimers is expected in all cases to be below the diffraction limit. This is consistent with our observations.

As one can see from the Table 5 and Figure 1 - figure supplement 3H,I a low stall force and a low unbinding rate yield similar theoretical curves as higher stall force and higher unbinding rate. Our model predicts that in a stalled regime, the lifetime of a kinesin of a microtubule is reduced from 11 to less than 0.3 seconds.

Based on currently available data our model cannot predict the magnitude of the stall force for Cik1-Kar3 kinesin. All values within the plausible rage (0.1 – 6 pN) result in good fits to experimental data given the accuracy of our data.
