# Supplementary material for: Non-catalytic motor domains enable processive movement and functional diversification of the kinesin-14 Kar3
Source: eLife. 2015 Jan 27;4:e04489. doi: 10.7554/eLife.04489 (PMC4338441; doi:10.7554/eLife.04489)
Supplement: Supplementary file 2. — This file contains tables listing the yeast strains and plasmids used in this study. DOI: http://dx.doi.org/10.7554/eLife.04489.024 [file elife04489s002.docx]

**Supplementary File 2**

**Table S6. Yeast strains used in this study**

**Strain name *genotype***

| CMY357 | *MAT α, leu2-3,112, hisΔ200::Kar3-3xGFP::HIS3, pHIS3::mCherry-Tub1::URA3, Cik1^WT^-6xHA::KanMX* |
| --- | --- |
| CMY358 | *MAT α, leu2-3,112, hisΔ200::Kar3-3xGFP::HIS3, pHIS3::mCherry-Tub1::URA3, Cik1^1-521^-6xHA::KanMX* |
| CMY359 | *MAT α, leu2-3,112, hisΔ200::Kar3-3xGFP::HIS3, pHIS3::mCherry-Tub1::URA3, Cik1^1-360^-6xHA::KanMX* |
| CMY270 | *MAT α, leu2-3,112, hisΔ200::Kar3-3xGFP::HIS3, pHIS3::mCherry-Tub1::URA3* |
| CMY214 | *MAT α, hisΔ200, leu2-3,112, ura3-52, lys2-801am, Δcik1::KanMX* |
| DDY1810 | *MAT a; leu2, ura3-52, trp1, prb1-1122, pep4-3, pre1-451* |
| CMY120 | *MAT a; leu2, ura3-52, trp1, prb1-1122, pep4-3, pre1-451, CIK1-6xHis6xFlag::KanMX* |
| KDY3 | *MAT a; ura3-52, trp1, prb1-1122, pep4-3, pre1-451, CIK1-6xHis6xFlag::KanMX, KAR3-eGFP::LEU2* |
| CMY107 | *MAT a; leu2, ura3-52, trp1, prb1-1122, pep4-3, pre1-451, VIK1-6xHis6xFlag::KanMX* |
| CMY290 | *MAT α, leu2-3,112, ura3-52, lys2-801am, hisΔ200::Kar3-3xGFP::HIS3, Δcik1::KanMX* |
| CMY202 | *MAT a; lys2-801am, cdc20::TRP1::GAL1/10-CDC20, hisΔ200::Kar3-3xGFP::HIS3, pHIS3::mCherry-Tub1::URA3, Δcik1::KanMX* |
| CMY203 | *MAT a; lys2-801am, cdc20::TRP1::GAL1/10-CDC20, hisΔ200::Kar3-3xGFP::HIS3, pHIS3::mCherry-Tub1::URA3, Δvik1::KanMX* |
| CMY197 | *MAT a, leu2-3,112, lys2-801am, hisΔ200::Kar3-3xGFP::HIS3, pHIS3::mCherry-Tub1::URA3, Δbim1::KanMX* |
| CMY326 | *MAT a, leu2-3,112, hisΔ200::Kar3-3xGFP::HIS3, pHIS3::mCherry-Tub1::URA3, Δbik1::URA3,* |
| CMY371 | *MAT a, his3∆200, promURA3::tetR::GFP-LEU2, tetOx112-URA3, Cik1^WT^-6xHA::KanMX* |
| CMY372 | *MAT α, his3∆200, promURA3::tetR::GFP-LEU2, tetOx112-URA3, Cik1^1-360^-6xHA::KanMX* |
| CMY373 | *MAT α, his3∆200, promURA3::tetR::GFP-LEU2, tetOx112-URA3, Cik1^1-521^-6xHA::KanMX* |
| CMY381 | *MAT α, his3∆200, lys2-801am, ura3-52, Cik1^1-353^Vik1^351-647^-Flag::LEU2* |
| CMY382 | *MAT α, his3∆200, lys2-801am, ura3-52, Cik1^WT^-Flag::LEU2* |
| CMY383 | *MAT α, his3∆200, lys2-801am, GFP-Tub1::URA3, Cik1^1-353^Vik1^351-647^-Flag::LEU2* |
| CMY384 | *MAT α, his3∆200, lys2-801am, GFP-Tub1::URA3, Cik1^WT^-Flag::LEU2* |

**Table S7. Plasmids used in this study**

**Plasmid**

**Name Description**

| pCM107 | CIK1-Flag (NotI/ClaI) + KAR3 (SalI/KpnI) in pESC-TRP |
| --- | --- |
| pCM136 | CIK1^35-594^-Flag (NotI/ClaI) + KAR3 (SalI/KpnI) in pESC-TRP |
| pCM137 | VIK1-Flag (NotI/ClaI) + KAR3 (SalI/KpnI) in pESC-TRP |
| pCM140 | CIK1^250-594^-Flag (NotI/ClaI) + KAR3 (SalI/KpnI) in pESC-TRP |
| pCM176 | CIK1-Flag (NotI/ClaI) + Halo (BamHI/ApaI)-KAR3 (SalI/KpnI) in pESC-TRP |
| pCM177 | VIK1-Flag (NotI/ClaI) + Halo (BamHI/ApaI)-KAR3 (SalI/KpnI) in pESC-TRP |
| pCM191 | CIK1^35-594^-Flag (NotI/ClaI) + Halo (BamHI/ApaI)-KAR3 (SalI/KpnI) in pESC-TRP |
| pCM192 | CIK1^250-594^-Flag (NotI/ClaI) + Halo (BamHI/ApaI)-KAR3 (SalI/KpnI) in pESC-TRP |
| pCM206 | KAR3-Flag (NotI/SacI) + Halo (BamHI/ApaI)-KAR3 (SalI/KpnI) in pESC-TRP |
| pCM210 | CIK1-Flag (NotI/ClaI) + Halo (BamHI/ApaI)-eGFP (ApaI/SalI)-KAR3 (SalI/KpnI) in pESC-TRP |
| pCM220 | CIK1^1-360^-Flag (NotI/ClaI) + Halo (BamHI/ApaI)-KAR3 (SalI/KpnI) in pESC-TRP |
| pCM221 | CIK1^1-521^-Flag (NotI/ClaI) + Halo (BamHI/ApaI)-KAR3 (SalI/KpnI) in pESC-TRP |
| pCM227 | CIK1-Flag (NotI/ClaI) + Halo (BamHI/ApaI)-KAR3^70-729^ (SalI/KpnI) in pESC-TRP |
| pCM228 | CIK1-Flag (NotI/ClaI) + Halo (BamHI/ApaI)-KAR3^116-729^ (SalI/KpnI) in pESC-TRP |
| pCM229 | CIK1-Flag (NotI/ClaI) + Halo (BamHI/ApaI)-KAR3^174-729^ (SalI/KpnI) in pESC-TRP |
| pCM230 | CIK1-Flag (NotI/ClaI) + Halo (BamHI/ApaI)-KAR3^277-729^ (SalI/KpnI) in pESC-TRP |
| pCM231 | KAR3-Flag (NotI/SacI) + Halo (BamHI/ApaI)-KAR3^G479E^ (SalI/KpnI) in pESC-TRP |
| pCM270 | CIK1^1-353^ VIK1^351-647^-Flag (NotI/ClaI) + Halo (BamHI/ApaI)-KAR3 (SalI/KpnI) in pESC-TRP |
